# Yeast genomic expression patterns in response to low-shear modeled microgravity

**DOI:** 10.1186/1471-2164-8-3

**Published:** 2007-01-03

**Authors:** Kathy B Sheehan, Kate McInnerney, Boloroo Purevdorj-Gage, Sara D Altenburg, Linda E Hyman

**Affiliations:** 1 Division of Health Sciences, Montana State University, Bozeman, MT 59717, USA; 2 Department of Microbiology, Montana State University, Bozeman, MT 59717, USA

## Abstract

The low-shear microgravity environment, modeled by rotating suspension culture bioreactors called high aspect ratio vessels (HARVs), allows investigation in ground-based studies of the effects of microgravity on eukaryotic cells and provides insights into the impact of space flight on cellular physiology. We have previously demonstrated that low-shear modeled microgravity (LSMMG) causes significant phenotypic changes of a select group of Saccharomyces cerevisiae genes associated with the establishment of cell polarity, bipolar budding, and cell separation. However, the mechanisms cells utilize to sense and respond to microgravity and the fundamental gene expression changes that occur are largely unknown. In this study, we examined the global transcriptional response of yeast cells grown under LSMMG conditions using DNA microarray analysis in order to determine if exposure to LSMMG results in changes in gene expression.

LSMMG differentially changed the expression of a significant number of genes (1372) when yeast cells were cultured for either five generations or twenty-five generations in HARVs, as compared to cells grown under identical conditions in normal gravity. We identified genes in cell wall integrity signaling pathways containing MAP kinase cascades that may provide clues to novel physiological responses of eukaryotic cells to the external stress of a low-shear modeled microgravity environment. A comparison of the microgravity response to other environmental stress response (ESR) genes showed that 26% of the genes that respond significantly to LSMMG are involved in a general environmental stress response, while 74% of the genes may represent a unique transcriptional response to microgravity. In addition, we found changes in genes involved in budding, cell polarity establishment, and cell separation that validate our hypothesis that phenotypic changes observed in cells grown in microgravity are reflected in genome-wide changes.

This study documents a considerable response to yeast cell growth in low-shear modeled microgravity that is evident, at least in part, by changes in gene expression. Notably, we identified genes that are involved in cell signaling pathways that allow cells to detect environmental changes, to respond within the cell, and to change accordingly, as well as genes of unknown function that may have a unique transcriptional response to microgravity. We also uncovered significant changes in the expression of many genes involved in cell polarization and bud formation that correlate well with the phenotypic effects observed in yeast cells when grown under similar conditions. These results are noteworthy as they have implications for human space flight.

## Background

Spaceflight presents a novel environment for organisms that evolved under the selection pressures of gravity on Earth. Cellular responses to the environment are mediated by phenotypic changes ultimately driven by alterations in gene expression, some of which resemble environmental stress responses [1,2]. Microorganisms such as yeast serve as powerful eukaryotic models for experimentally investigating the effects of microgravity. Studies conducted with microorganisms have shown that cells sense and respond to changes in mechanical forces, such as gravity, with changes in microbial growth and behavior, including a shortened lag phase, increased growth rates, increased resistance to antibiotics [3,4], and changes in transcriptional response [5]. However, comprehensive understanding of the effects of microgravity on biological systems is difficult due to technical problems associated with spaceflight experiments. Many factors that can influence cell physiology are present during spaceflight, such as exposure to hypergravity during launching, radiation, disturbances in circadian rhythms, physical stresses, and changes in nutritional uptake [6], in addition to microgravity. Utilizing the yeast model system to isolate one of the variables, allows an investigation of the cellular response specific to microgravity conditions. We employed, in our study, a specialized ground-based bioreactor, called the High Aspect Ratio Vessel (HARV), which closely mimics the microgravity environment and provides an efficient means to study the effects of weightlessness on yeast cells [3,7,8].

The HARV is a rotating wall culture vessel that reduces fluid shear. Cells are inoculated into the HARV and air bubbles removed so as not to disturb convection currents produced in the rotating suspension. In this vesicle the cells revolve around a horizontal axis (parallel to the ground and perpendicular to the gravitational force vector), continuously falling through the fluid at 1 g under terminal velocity conditions. Experimental control conditions are achieved by orienting the HARV so that cells revolve around a vertical axis (see Figure 1 in [3]). Cells are not agitated, but move sufficiently enough in the HARV to allow for the continuous exchange of dissolved gases through a permeable membrane in the device and for exchange of nutrients and wastes in the medium within the vessel. In addition, the system randomizes the unidirectional pull of Earth's gravity and minimizes turbulence over the surface of the cells creating the net affect of a state of 'functional weightlessness' or 'low-shear modeled microgravity' (LSMMG) [8,9]. The bioreactor does not remove the force due to gravity, but rather the gravitational vector present in the rotating device constantly changes direction (vector averaged gravity) such that cells remain suspended in solution [8]. Thus, the HARV bioreactor, which mimics some of the same conditions of low-shear encountered during space flight, serves as a ground-based analog for space experiments [10-12].

While numerous environmental signals have been examined in modeled microgravity conditions for their effects on bacteria [3,12], few studies have utilized yeast. *S. cerevisiae *is fully viable and responsive to microgravity conditions either during spaceflight or in ground-based experiments [3]. Our initial examination of gene expression in yeast cells exposed to LSMMG conditions provided some of the first lines of evidence that microgravity influences certain aspects of phenotypic and behavioral traits in yeast [1] and showed that growth in HARVs might be regulated transcriptionally by activation through two promoter elements, one with direct ties to a well-studied signal transduction pathway. Johanson et al. [2] expanded the initial gene expression study in *S. cerevisiae *and identified significant genes with changes in gene expression in response to low-shear conditions [1,2].

In a recent study in our laboratory, we examined the morphological changes of yeast cells grown in LSSMG compared to planktonic growth. We observed defective polarity determination as exhibited by aberrant (random) budding compared to the typical bipolar pattern of controls. We postulated that the random budding pattern observed in cells exposed to LSMMG might be the result of changes in expression of genes that play roles in upstream budding processes. For example, processes such as bud site selection and polarity establishment may be pathways that are disrupted when the gravitational sense of cells is neutralized, as is the case during HARV rotational growth. Our results confirmed that a select group of genes associated with the establishment of cell polarity, bipolar budding, and cell separation were significantly altered, suggesting that low-shear conditions alter yeast gene expression in evolutionarily conserved cellular functions such as cell polarization [13].

Our current hypothesis is that *S. cerevisiae *will undergo a transcriptional response to growth conditions that reflect the space environment and that this response will be evident as changes in expression of specific genes, including those involved in cell polarity, budding, and mother-daughter cell separation, as well as genes that participate in the cellular response to environmental stress. We utilized microarray analysis and comparative genomics to directly assess and quantify global gene expression patterns in cultures grown under LSMMG. We identified a general transcriptional response of *S. cerevisiae *to microgravity, as well as effects of longer exposure of cells grown under continuous culture conditions. Our results reveal the ways in which microgravity affects yeast cells, and provide insights into the fundamental mechanisms controlling establishment of cell polarity and polarity dependent cellular responses.

## Results and discussion

### Low-shear modeled microgravity

*S. cerevisiae *cells grown in HARV bioreactors in our study show a dramatic genome-wide transcriptional response to low-shear modeled microgravity when compared to cells grown in HARVs under control conditions. To assure that gene expression changes could not be attributed to a response to the diauxic shift [14], cells were incubated under LSMMG culture conditions or in control culture conditions for 5 or 25 generations and maintained in mid-log phase by repeated serial dilution (see Methods). We also monitored the metabolic state of the cells by measuring the relative glucose levels, that showed no dramatic decreases in the control culture samples compared to experimental cultures in stationary phase growth (mean values ΔA_540 _= Control 5 generations = 0.794 ± 0.072; Experimental 5 generations = 0.666 ± 0.056; Control 25 generations = 1.1.618 ± 0.073 ; Experimental 25 generations = 1.636 ± 0.078) confirming that samples used for hybridization were in logarithmic growth phase.

### Global microgravity response

**Figure 1:**
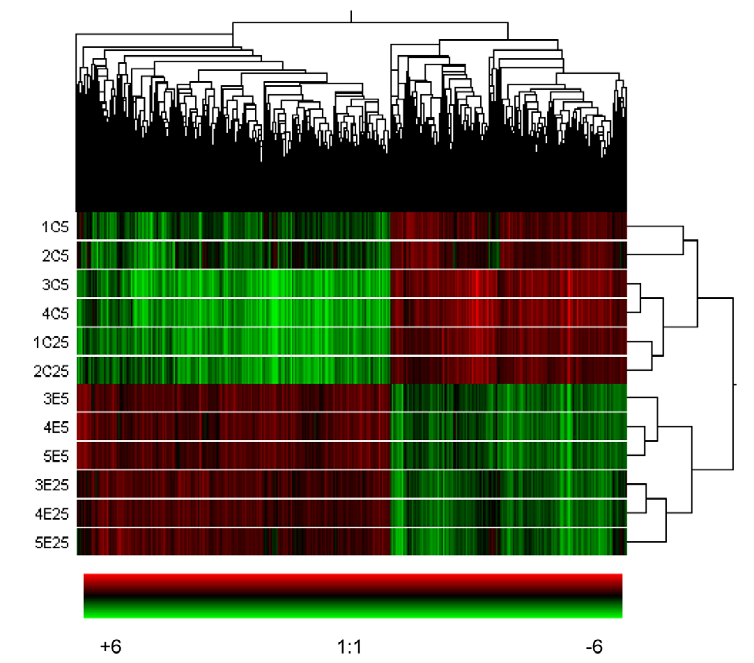
**Gene expression response to low-shear modeled microgravity**. Two-dimensional hierarchical cluster heat map analysis of the microarray transcriptional profile of gene response to low-shear modeled microgravity (LSMMG) in *S. cerevisiae *wild-type diploid cells (BY4743) grown in HARVs (experimental group) for 5 generations (3E5, 4E5, 5E5) or 25 generations (3E25, 4E25, 5E25) versus the control group grown in HARVs for 5 generations (1C5, 2C5, 3C5, 4C5) or 25 generations (1C25, 2C25). The analysis was performed on 1372 statistically significant genes (from the filtered list of 3850 genes) with a fold change of at least 2 in one of three comparisons as described in *Methods*. Levels of expression are indicated on a color scale where red represents the up-regulated genes and green, the down-regulated genes. Similarities or differences in the experimental versus control gene expression patterns are represented in the horizontal dendrogram. The vertical dendrogram shows similarities in expression patterns between cultures exposed to LSMMG versus control cultures.

Using the Affymetrix gene chip system, we performed expression analysis of yeast subjected to growth in low-shear modeled microgravity. Of 3,850 normalized and filtered genes (see Methods), 1372 (36%) were significantly altered by exposure to LSMMG (approximately 2 fold changes were included in the data set). The list of genes and fold change values are not shown but are available from NCBI Gene Expression Omnibus: GSE4136 [15]. Hierarchical cluster analysis shows the overall transcriptional response of HARV-grown cells that have or have not been subjected to LSMMG (Figure 1).

Genes whose expression patterns changed during growth in LSMMG compared to controls grown in HARVs under normal gravity conditions (see Methods) were classified into major functional categories (Supplemental Information, Table 1; Figure 1) according to the Munich Information Center for Protein Sequences (MIPS) Comprehensive Yeast Genome Database (CYGD) [13,16]. Additional File 1 shows the numbers of up regulated (induced) and down regulated (repressed) genes as well as the percentage of genes in each category affected by LSMMG. Numerically, the top five categories are metabolism, unclassified, transcription, protein with binding function, and protein fate. Notably, one of the genes most dramatically affected by LSMMG is HSP30 (fc = 13.23, up regulated) which is known to be responsive to heat, ethanol, weak acid exposure and high hydrostatic pressure [20-22].

We also determined the global response of log-phase yeast to continuous culture in LSMMG as a function of generation time. HARV-grown cells were harvested after 5 and 25 generations of growth. Although the numbers and types of genes in specific functional categories varied somewhat, short- and long-term cultures were remarkably similar with respect to which genes were up- or down-regulated [See Additional File 2]. The set of genes whose expression-level was significantly altered at 5 generations, relative to the control, was, for the most part, the same set whose expression was significantly altered at 25 generations (278 genes were significant at 5 generations. Of these, 161 genes were up regulated and 117 genes were down-regulated. 197 genes were significant at 25 generations. Of these, 106 were up-regulated and 91 were down-regulated. 897 genes did not differ between 5 and 25 generations. Of these, 513 were up-regulated and 384 were down-regulated); Previous microarray analyses of yeast growing in LSMMG also showed expression changes, although the total number of genes involved was much less than we observed [1,2]. In a related study, Johanson et al. [2] identified 140 differentially expressed genes in which a significant number of genes are involved in catalytic activity. Few are the same as genes identified in our experiment. Differences in strain genotypes, experimental growth conditions, and mode of microarray analysis make comparing the two studies problematic.

Function unknown (FUN) genes ranked as the most numerically abundant category of genes whose expression significantly changed under simulated microgravity. Aside from FUN genes, genes in the "metabolic" functional category comprise the next most abundant class of genes significantly altered by microgravity. This response may be due to the unique, altered fluid dynamics of the microenvironment surrounding cells during growth in LSMMG [8,9]. The HARV bioreactor minimizes mechanical stresses on cell aggregates in culture by producing a laminar flow of culture medium with subsequent low shear and turbulence in an optimized suspension culture. However, both low shear and turbulence are affected by the terminal velocity of the yeast cells in the medium and the diffusion of nutrients into the cells, processes that are influenced by cell size, the availability of the nutrients, the speed of rotation of the HARVs, buoyancy, and the viscosity of the culture medium [7-9]. It seems likely that the microgravity fluid dynamics in our experiment caused the large metabolic response we observed in the microgravity array dataset compared to controls.

### LSMMG compared to other environmental stress responses

**Figure 2:**
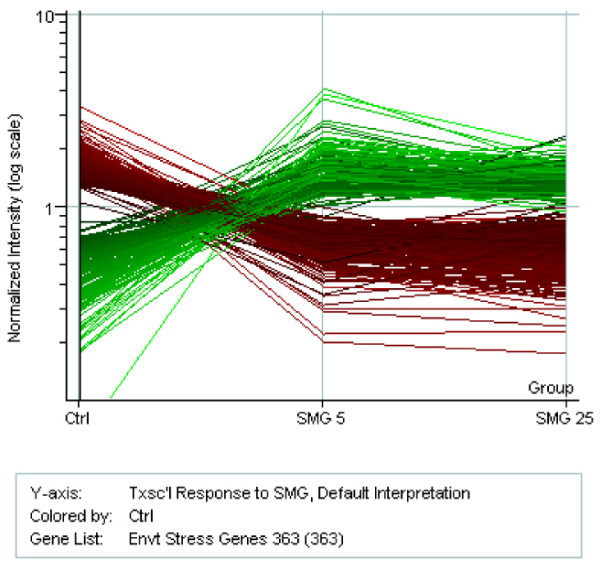
A comparison of the low-shear modeled microgravity response (SMG) with a previously identified list of 868 *S. cerevisiae *Environmental Stress Response (ESR) genes with a fold change >2 identified by Gasch et al. (2002). 363 genes from our dataset change > 2-fold under all conditions.

**Figure 3:**
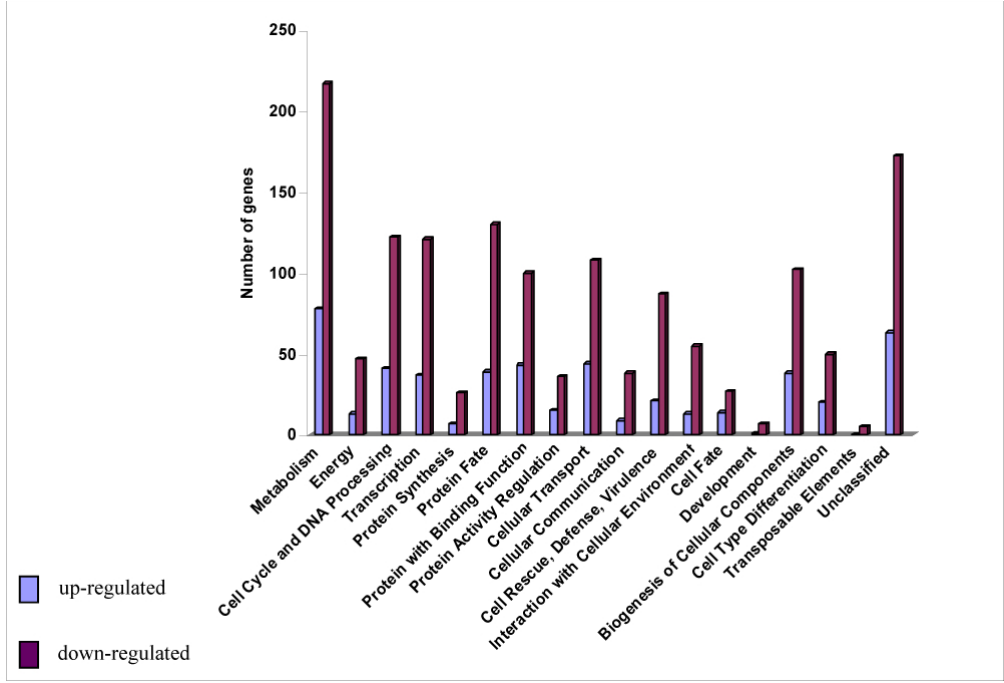
IPS functional classification of the list of 1006 significant genes that respond to LSMMG that are not general environmental stress response genes as identified by Gasch et al., 2000. Black bars represent genes that are up-regulated, gray bars represent the down-regulated genes.

Cells continually face environmental challenges, and therefore have evolved both general, and stress specific pathways to meet these challenges. Microarray analyses have done much to illuminate these pathways. [21,23-26]. One such study by Gasch et al.; 2000, defined a set of ~ 900 genes from *S. cerevisiae*, that always responded to any stress. They defined this response as the "environmental stress response" (ESR). Reasoning that microgravity might represent an environmental stress, we compared data in our experiment to the dataset of Gasch et al (Figure 2). This comparison revealed that 26% of the genes that responded to LSMMG could be defined as known ESR genes. The remaining 74% may represent a unique response to microgravity. We functionally categorized these genes using MIPS (Figure 3).

Notably, the concept that cells respond to the effects of microgravity with a generalized stress response is supported by studies by Lynch et al. [27] who demonstrated that simulated microgravity increases a generalized stress resistance to hyperosmosis and low pH in *E. coli *cells and imparts superresistance to the cells. In addition, work conducted using *Salmonella*, showed that cells became more robust in microgravity and more potent as pathogens [28]. Indeed, Wilson et al. [5] identified a stress specific subset of genes in a study conducted in HARVs using *Salmonella enterica *and proposed that several genes in their analysis, might identify a LSMMG 'regulon'. LSMMG clearly alters expression of yeast genes with diverse function although we could not draw any inferences about whether these genes comprise a yeast-specific 'regulon'. However, when our dataset was analyzed for common regulatory motifs (GeneSpring) the only consensus sequence identified was the STRE (stress responsive element) which was present in the majority of genes that were up-regulated (72%) but not in the genes that were down-regulated in response to microgravity. While many of the genes upregulated in response to microgravity present in our study are common to those identified by Gasch et. al., there is a group that contains the STRE that is not included in the ESR, suggesting that theses genes may be responding to the unique to the pressures of LSMMG.

### Budding and polarity response

**Figure 4:**
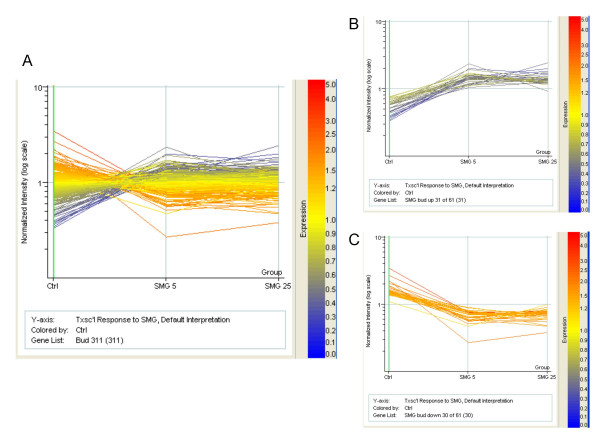
**A. **A comparison of the low-shear modeled microgravity response (SMG) with 311 MIPS functional category (43.01.03.05) genes involved in budding and cell polarity functions. **B. **The 31genes from the list of 311 that are up-regulated greater than 2-fold compared to controls. **C. **The 30 genes from the list of 311 that are down-regulated greater than 2-fold compared to controls. Expression levels are indicated on the color scale bars.

The mechanisms that control polarized cell divisions are just beginning to be understood at the genome-wide level. How cells determine which way is "up" is certain to influence patterns of cell growth and division [17,18]. Understanding how yeast cells establish polarity may contribute to our understanding of how similar processes occur in higher eukaryotes. Normal yeast cells do not assemble buds at random on the cell surface. Rather cells respond to cortical cues and obey a program that promotes polarized growth by establishing bud sites in a stereotypical fashion. Our laboratory has studied the phenotypic response of cells to simulated microgravity, and observed striking changes in the establishment of cell polarity, bipolar budding pattern, and cell separation [13]. These phenotypic changes are likely driven by specific changes in the expression of proteins involved in polarity-dependent pathways. Previous work in our laboratory demonstrated that cells exposed to LSMMG showed random bud patterns [13]. Therefore, we compared the 1372 genes significantly affected by LSMMG to genes in the MIPS "budding and cell polarization" classification category (Figure 4). 20% of the LSMMG genes matched genes identified by MIPS (Panel A). Equal numbers of genes are up-regulated (Panel B) and down-regulated (Panel C). Table 1 shows up and down fold changes for thirty-one genes with a biological description matching 'budding and cell polarity'.

Ni and Snyder performed an experiment in which they screened 4168 members of the homozygous diploid yeast deletion collection for nonessential genes involved in bipolar bud site selection [19]. They identified 127 genes which when deleted altered wild-type budding patterns. Of these, 104 were represented in our study. 2/3 of these were significant in our study, with 14 genes having a fold change of at least 2 (Table 1). In our earlier study we determined that several of these genes were affected by growth in LSMMG [13]. Specifically, four are involved in bud pattern selection (BUD5, RAX1, RAX2, and BUD25) and three are involved in mother-daughter cell separation [DSE1, DSE2, and EGT2). These are shown in Table 1. In the present study, the genes showed the same trends in up- and down-regulation. Taken together, the data support our hypothesis that genes that effect polarity underlie microgravity-induced changes in cells.

Furthermore, in our previous study, in addition to polarity determination, we also observed a random budding phenotype in response to LSMMG [13]. Data derived from our microarray analyses support this observation. For example LSSMG grown cells showed diminished NSR1 expression (fc = 5.6). The homozygous diploid NSR1 deletion strain exhibits a strong random budding phenotype [29]. NSR1 gene products affect rRNA processing and may function in bud site selection through ribosome biogenesis or translation. We also observed strong down regulation of the ribosomal protein genes, RPL22A (fc = 3.8), and RPS18B (fc = 2.6), the lipid metabolism gene FEN1 (fc = 4.1), and BUD21 (fc = 2.6). Interestingly, when BUD21, a gene that encodes a protein of unknown function, is deleted cells exhibit a random budding phenotype [29].

Finally, genes that exhibit bud-specific localization are affected by LSMMG. Notably, 11 of the 24 (46%) bud-localized transcripts were significantly altered. These include EGT2 (fc = 7.7, down-regulated), ASH1 (fc = 1.75, down-regulated), TPO1 (fc = 2.1 down-regulated), and WSC2 (fc = 2.0 down-regulated). The functions of some of the genes may hint at mechanistic pathways that are LSMMG sensitive, including WSC2 that encodes a heat shock sensor which transduces signals via a MPK1 pathway [31], EGT2, a gene involved in cell separation [32], and ASH1, a daughter cell-specific transcription factor [30].

### Cell wall integrity and potential sensing mechanisms

Yeast cells must maintain cell wall integrity when challenged by rapid and extreme changes in the extracellular environment induced by prolonged exposure to heat, osmotic stress, and/or oxidative stress [31, 36]. In addition, during polarized cell growth, such as budding, cells must maintain cell shape and prevent rupture during the formation of a bud and subsequent cell division. The highly regulated cell wall integrity (CWI) MAPK signaling pathway is primarily responsible for responding to external cell wall stress and for coordinating changes to the cell wall [35]. Notably, in our study, SDP1 (YIL113w) was up-regulated (fold change of 6.71 at 5 generations and 8.12 at 25 generations. Sdp1 is a stress inducible dual-specificity MAP kinase phosphatase that negatively regulates the MAP kinase cell integrity pathway [33, 34, 35]. An increase in SDP1 in yeast cells under stress from LSMMG, detected in our microarray experiment, and subsequent down regulation of the MAP kinase cascade, could lead changes in cell shape and polarity. In addition, we found an increase in expression of PTP2 (fc = 2.5, up regulated), a protein tyrosine phosphatase, that also down-regulates the MAP kinase pathway [36]. Considered together with the changes in phenotype for cells exposed to simulated microgravity, our results suggest that LSMMG influences multiple signaling pathways. The changes in phenotype, together with the microarray results, implicate the cell wall integrity MAP kinase pathway as a possible mechanism for sensing the low shear microgravity environment.

## Conclusion

The microarray data presented herein provide insights to the genomic response of yeast to microgravity. Ultimately, yeast microarray analysis could help to determine how spaceflight might alter gene expression in humans. Our data shows that a large fraction of the yeast genome responds early to simulated microgravity and that the changes persist for 25 generations. We identified genes that are involved in cell signaling pathways that may enable cells to detect and respond to the LSMMG. Previously, we reported that microgravity affects cell polarity and bud formation [13]. The transcriptome profiling of cells exposed to LSMMG shows coordinated regulation of genes that control polarity establishment. Future studies will be geared toward identifying the molecular signatures that drive the LSMMG, as a first step towards unraveling what are likely to be a complex but important mechanisms allowing life to exist in extreme environments such as space.

## Methods

### Yeast strains and growth conditions

*Saccharomyces cerevisiae *diploid strain BY4743 (MAT A/á 4741/4742) obtained from Invitrogen (Carlsbad, CA) was grown overnight at 30°C with shaking at 250 rpm in rich medium (YPD broth, Sigma, St Louis) to OD_600 _= 1.5–2.0. Aliquots of the cultures were used to inoculate fresh 60 ml YPD broths to an initial OD_600 _= ~.075 and the cells were loaded into HARVs. The HARV cultures initially were allowed to recover for approximately 4 hours and OD_600 _readings were monitored until the cultures entered the logarithmic growth phase. Growth in low-shear modeled microgravity (LSMMG) was achieved by culturing cells at 30°C in the vertical position in HARV bioreactors rotating at 30 rpm. Control cultures were set up identically to the LSMMG HARVs, with one exception. The control HARVs were placed in the horizontal position in the bioreactors [3, 7, 37].

In order to prevent cells from entering stationary phase, throughout the experiment, we performed batch dilutions by monitoring optical density and diluted cultures (1:10,000) at approximately every five generations (10 hours). We performed the batch dilutions at 5, 10, 15, and 20 doublings as follows. Cultures were removed from the HARVs while in mid-log phase, OD_600 _readings were recorded, and aliquots of the cultures were used to inoculate fresh YPD broths that were loaded in the HARVs. The remaining cultures were pelleted by centrifugation at 5000 X g for 5 min at room temperature. Aliquots of the supernatants were frozen at -20°C for later analysis of glucose concentration. The cell pellets were flash frozen in dry ice/ethanol and stored at -80°C until further processing.

### Sample collection, cell lysis, glucose measurement, and RNA isolation

Media glucose concentration was determined using the GO Assay Kit (Sigma, St Louis, MO) following instructions provided by the manufacturer. Each sample was run in duplicate.

Total RNA was extracted using the QIAGEN^®^RNeasy^®^Midi Kit (QIAGEN, Inc., Valencia, CA) according to the manufacturer's instructions with the following modification to the cell lysis step. We added 0.1 mm diameter sterile zirconia/silica beads (BioSpec Products, Inc., Bartlettsville, OK) to the yeast cells in buffer RLT, then bead beat in the Savant Fast Prep bead beater (Savant Instruments, Inc., Farmingdale, NY) at speed 6.5 for 45 seconds. Samples were immediately cooled on ice.

Samples for microarray analysis were further purified and concentrated using the QIAGEN^®^RNeasy^®^MinElute Cleanup Kit (QIAGEN, Inc., Valencia, CA). RNA quality was evaluated by 1.2% formaldehyde/agarose gel electrophoresis according to the QIAGEN protocol in Appendix H of the RNeasy^®^Mini Handbook. RNA concentrations and purity were determined by measuring absorbances at 260 nm and 280 nm on an Ultrospec GeneQuant (GE Healthcare) spectrophotometer.

### Microarray hybridization

Yeast RNA was hybridized to Affymetrix GeneChipYeast Genome 2.0 Arrays (Affymetrix, Santa Clara, CA) as described in the users' manual (Affymetrix GeneChip Expression Analysis Technical Manual, 2004), using the GeneChip Expression 3' Amplification One-Cycle Target Labeling and Control Reagents kit. Briefly, total RNA was reverse transcribed to cDNA using a T7-oligo (dT) primer. Following second-strand cDNA synthesis, the double-stranded cDNA was purified as a template for the subsequent in vitro transcription (IVT) reaction. Linearly amplified biotin-labeled complementary RNA (cRNA) was synthesized in the presence of a biotinylated nucleotide analog/ribonucleic acid mix. The labeled cRNA was purified, fragmented, and hybridized to the arrays at 45°C for 16 hours with constant rotational mixing at 60 rpm. Washing and staining of the arrays was performed using the Affymetrix GeneChip Fluidics Station 450. Arrays were scanned using an Affymetrix GeneChip Scanner 3000 and GCOS software.

### Data analysis

The data set was analyzed using GeneSpring software version 7.2 (Silicon Genetics, Palo Alto, CA). The Affymetrix CEL files were RMA normalized; genes were filtered for threshold signal intensities of at least 50 in 1 of three replicate conditions. Statistically significant differentially expressed genes were identified through ANOVA using the Welch t-test with a Benjamini & Hockberg test correction that predicts a false discovery rate of about 5% of the genes identified. The resulting list was further narrowed by comparing replicate sets to identify genes with fold change differences in expression of at least 2.0. For access to complete data sets, see NCBI Gene Expression Omnibus: GSE4136 [15]. Cells for three replicates of 'target' RNA extractions were collected at time points that correspond to 5 and 25 generations of growth from experimental and control HARVs in order to determine whether the yeast response to the stress was transient or long-term. Target RNA was hybridized to GeneChip Yeast Genome 2.0 Arrays (Affymetrix, Santa Clara, CA). Comparative clustering analysis showed that one of the control arrays did not cluster with the replicate control arrays (data not shown). The array had low background expression resulting in an aberrant expression pattern compared to all the other arrays. Therefore, the data from the aberrant array was deleted from subsequent statistical analyses. The remaining control arrays were combined into one group, which served as the control group (Ctrl) for further analysis. The three combined replicates each for 5 and 25 generations of growth in LSMMG were designated SMG 5 and SMG 25 respectively.

### Comparison of data with other *S. cerevisiae *stress data

We compared our data set with a list of 830 *S. cerevisiae *Environmental Stress Response Genes (ESR), obtained from the web site of Gasch et al. (2000) [38], using GeneSpring software and standard correlation.

## Authors' contributions

KBS designed and carried out the study, coordinated and participated in the data analysis, and wrote the manuscript. KM carried out all the Affymetrix fluidics station protocols and performed statistical analyses. P-GB participated in the design of the study, helped to interpret the results, and contributed to the manuscript. SDA participated in data interpretation and contributed to writing the manuscript. LEH conceived of the study, participated in the experimental design and analysis, contributed to the writing of the manuscript and provided guidance with the manuscript. All authors read and approved the final manuscript.

## Supplementary Material

Additional file 1 Microgravity Response to Generation Time. List of functional categories and subcategories activated by exposure to low-shear modeled microgravity based on MIPS classification. The proportions of up-regulated and down-regulated genes are listed. The top five subcategories are shown in bold type. Click here for file

Additional file 2 Microgravity Response to Generation Time. The global functional classification of the 1372 genes affected by growth in LSMMG. The graph shows the distributions of the Early (Short-term) Response genes, each of which is subdivided into the up- and down- regulated genes and the Late (Long-term) Response genes, subdivided into the up- and down- regulated genes. The classification is based on the MIPS database available on the web.
Click here for file

## Figures and Tables

**Table 1:** Direction and fold change of genes that establish cell polarity and bipolar budding in yeast cultured under simulated microgravity.

**Gene**	**Genbank ID**	**Fold Change 5 generations v. ctrl**	**Fold Change 25 generations v. ctrl**	**Description**
**Genes in the Purevdorj-Gage study [13]**				

RAX1	YCR301W	5.3 ↓	5.1 ↓	bipolar budding
RAX2	YLR084C	3.2 ↓	3.4 ↓	bipolar budding
DSE1	YER124C	8.6 ↓	6.0 ↓	cell separation
DSE2	YHR143W	3.1 ↓	3.1 ↓	cell separation
BUD5	YCR038C	1.7 ↑	1.6 ↑	polarity
BUD25	YER014C-A	1.6 ↑	1.7 ↑	bud site selection
EGT2	YNL327W	7.7 ↓	6.1 ↓	cell separation

**Bipolar budding pattern genes identified in Ni and Snyder study [19]**				

SLG1	YOR008C	2.2 ↑	1.6 ↑	cell wall integrity, stress response
VMA5	YKL080W	2.1 ↓	1.5 ↓	transport
PRS3	YHL011C	2.2 ↓	2.0 ↓	regulation of cell size
BUD21	YOR078W	2.3 ↓	2.6 ↓	processing
SUR4	YLR372W	2.4 ↓	2.5 ↓	lipid metabolism
RSR1	YGR152C	2.5 ↓	2.0 ↓	bud site selection, cell polarity
RSP18B	YML026C	2.6 ↓	3.1 ↓	ribosomal protein
RPL22A	YLR061W	3.8 ↓	4.4 ↓	ribosomal protein
FEN1	YCR034W	4.1 ↓	3.2 ↓	lipid metabolism
NSR1	YGR159C	5.6 ↓	7.5 ↓	nuclear protein
GAS1	YMR307W	1.8 ↓	2.0 ↓	cell wall
BUD22	YMR014W	1.9 ↓	2.0 ↓	bud site selection
BUD23	YCR047C	1.8 ↓	2.4 ↓	bud site selection
STO1	YLR287C	2.1 ↓	1.7 ↓	protein biosynthesis
---	YLR287C	2.5 ↓	2.4 ↓	hypothetical ORF

**MIPS functional category: budding and cell polarity**				

ERV15	YBR210W	1.8 ↑	1.8 ↑	Axial bud site selection
BUD5	YCR038C	1.7 ↑	1.6 ↑	bud site selection
TWF1	YGR080W	1.5 ↑	1.6 ↑	bipolar bud site selection
BUD13	YGL174W	1.4↑	1.4 ↑	bud site selection
BUD20	YLR074C	1.2 ↑	1.1 ↑	bud site selection
MUB1	YMR100W	1.1 ↑	1.2 ↑	regulation of cell budding
BUD27	YFL023W	1.1 ↑	1.2 ↑	bud site selection
AXL1	YPR122W	1.1 ↑	1.1 ↑	bud site selection
THP1	YOL072W	1.1 ↑	1.2 ↑	bud site selection
LAS1	YKR063C	1.1 ↑	1.2 ↑	budding cell bud growth
STE20	YHL007C	1.0 ↓	1.0 ↓	bud site selection
BUD16	YEL029C	1.1 ↓	1.0 ↓	bud site selection
BUD7	YOR299W	1.2 ↓	1.1 ↑	bud site selection
BUD2	YKL092C	1.2 ↓	1.1 ↓	bud site selection
BUD8	YLR353W	1.2 ↓	1.3 ↓	bud site selection
BUD32	YGR262C	1.2 ↓	1.3 ↓	bud site selection
BUD17	YNR027W	1.2 ↓	1.0 ↓	bud site selection
RRP14	YKL082C	1.3 ↑	1.4 ↓	cell polarity
BUD3	YCL014W	1.3 ↓	1.4 ↓	bud site selection
BUD31	YCR063W	1.4 ↓	1.3 ↓	bud site selection
BUD6	YLR319C	1.4 ↓	1.4 ↓	bud site selection
AXL2	YIL140W	1.4 ↓	1.4 ↓	bud site selection
HKR1	YDR420W	1.5 ↓	1.7 ↓	bud site selection
BUD9	YGR041W	1.6 ↓	1.9 ↓	bud site selection
TOS2	YGR221C	1.6 ↓	1.7 ↓	budding cell bud growth
BUD23	YCR047C	1.8 ↓	2.4 ↓	bud site selection
BUD22	YMR014W	1.9 ↓	2.0 ↓	bud site selection
RSR1	YGR152C	2.5 ↓	2.0 ↓	bud site selection
BUD4	YJR092W	2.7 ↓	2.0 ↓	bud site selection
RAX2	YLR084C	3.2 ↓	3.2 ↓	bud site selection
RAX1	YOR301W	5.3 ↓	5.1 ↓	bud site selection
