# Supplementary material for: Yeast genomic expression patterns in response to low-shear modeled microgravity
Source: BMC Genomics. 2007 Jan 3;8:3. doi: 10.1186/1471-2164-8-3 (PMC1774566; doi:10.1186/1471-2164-8-3)
Supplement: Additional file 1 — Microgravity Response to Generation Time. List of functional categories and subcategories activated by exposure to low-shear modeled microgravity based on MIPS classification. The proportions of up-regulated and down-regulated genes are listed. The top five subcategories are shown in bold type. [file 1471-2164-8-3-S1.doc]

**Supplemental Table 1.**

|  |  |  |  |  | **Up- regualted genes** |  |  | **Down- regulated genes** |  | **Totals** |
| --- | --- | --- | --- | --- | --- | --- | --- | --- | --- | --- |
|  |  |  |  |  | **No. and**  **( %)** |  |  | **No. and (%)** |  |  |
|  |  | |  |  |  |  |  |  |  |  |
|  | Metabolism | |  |  |  |  |  |  |  |  |
| **Metabolism** | |  |  |  | **166 (24.7%)** |  |  | **210 (30.1%)** |  | **376** |
| Energy |  |  |  |  | 69  (10.2%) |  |  | 20  (2.87%) |  | 89 |
|  |  |  |  |  |  |  |  |  |  |  |
|  | Information Pathways | | |  |  |  |  |  |  |  |
| Cell Cycle & DNA processing | | |  |  | 68  (10.1%) |  |  | 127 (18.2%) |  | 195 |
| **Transcription** | |  |  |  | **85**  **(12.6%)** |  |  | **181**  **(26%)** |  | **266** |
| Protein Synthesis | |  |  |  | 9  (1.34%) |  |  | 95  (13.6%) |  | 104 |
| **Protein Fate** | |  |  |  | **125 (18.6%)** |  |  | **93**  **(13.3%)** |  | **218** |
| **Protein with Binding Function** | | | |  | **69**  **(10.2%)** |  |  | **161 (23.1%)** |  | **230** |
| Protein with Activity Regulation | | |  |  | 31  (4.61%) |  |  | 31  (4.46%) |  | 62 |
|  |  |  |  |  |  |  |  |  |  |  |
|  | Transport | |  |  |  |  |  |  |  |  |
| Cellular Transport | |  |  |  | 87  (12.9%) |  |  | 102 (14.6%) |  | 189 |
|  |  |  |  |  |  |  |  |  |  |  |
|  | Perception and Response to Stimuli | | | |  |  |  |  |  |  |
| Cell Communication/Signal Transduction | | | |  | 35  (5.21%) |  |  | 24  (3.44%) |  | 59 |
| Cell Rescue, Defense and Virulence | | | |  | 84  (12.5%) |  |  | 57  (8.18%) |  | 141 |
| Interaction with Cellular Environment | | | |  | 40  (5.96%) |  |  | 43  (6.17%) |  | 83 |
|  |  |  |  |  |  |  |  |  |  |  |
|  | Developmental Processes | | |  |  |  |  |  |  |  |
| Transposable Elements | | |  |  | 3  (0.44%) |  |  | 2  (0.28%) |  | 5 |
| Cell Fate |  |  |  |  | 21  (3.12%) |  |  | 32  (4.59%) |  | 53 |
| Development | |  |  |  | 6  (0.89%) |  |  | 6  (0.86%) |  | 12 |
| Biogenesis of Cellular Components | | | |  | 80  (11.9%) |  |  | 91  (13%) |  | 171 |
|  |  |  |  |  |  |  |  |  |  |  |
|  | Localization | |  |  |  |  |  |  |  |  |
| Cell Type Differentiation | | |  |  | 44  (6.55%) |  |  | 45  (6.46%) |  | 89 |
|  |  |  |  |  |  |  |  |  |  |  |
|  | Experimentally Uncharacterized | | |  |  |  |  |  |  |  |
| **Unclassified** | |  |  |  | **211 (31.4%)** |  |  | **93**  **(13.3%)** | | **304** |
